# Novel fluid biomarker detects TDP‐43 loss of function in individuals at elevated risk of Alzheimer’s disease

**DOI:** 10.1002/alz.094947

**Published:** 2025-01-09

**Authors:** Katherine E Irwin, Irika Sinha, Rachael E Wilson, Cynthia M. Carlsson, Henrik Zetterberg, Sterling C. Johnson, Jonathan P Ling, Philip C Wong

**Affiliations:** ^1^ Johns Hopkins University School of Medicine, Baltimore, MD USA; ^2^ Wisconsin Alzheimer’s Institute, University of Wisconsin School of Medicine and Public Health, Madison, WI USA; ^3^ Wisconsin Alzheimer’s Disease Research Center, University of Wisconsin School of Medicine and Public Health, Madison, WI USA; ^4^ Dementia Research Centre, Department of Neurodegenerative Disease, UCL Queen Square Institute of Neurology, University College London, London, United Kingdom, London United Kingdom; ^5^ Institute of Neuroscience and Physiology, University of Gothenburg, Gothenburg, Mölndal Sweden; ^6^ UK Dementia Research Institute at UCL, London United Kingdom; ^7^ Department of Medicine, University of Wisconsin‐Madison School of Medicine and Public Health, Madison, WI USA

## Abstract

**Background:**

TDP‐43 nuclear clearance and cytoplasmic aggregation occur in an estimated 30‐60% of cases of Alzheimer’s disease (AD), but this pathology can currently only be established at autopsy. Nuclear clearance of TDP‐43 leads to inclusion of cryptic exons in pre‐mRNA, some of which are spliced in‐frame and translated into proteins carrying novel cryptic exon‐encoded epitopes. We developed a Meso Scale Discovery (MSD) ELISA against the TDP‐43‐associated cryptic neoepitope within the HDGFL2 protein and found significantly elevated levels of this cryptic neoepitope in biofluids of presymptomatic ALS‐FTD (Irwin et al., *Nature Medicine*, 2024), indicating that cryptic HDGFL2 could serve as a biomarker for loss of TDP‐43 function. Here, we used our MSD assay to analyze TDP‐43 dysfunction in individuals at increased risk of AD due to family history.

**Method:**

A total of 266 CSF samples from 107 individuals (average age = 66.6 years, 40.8% phosphorylated‐tau181/Aβ42 ratio positive) in the Wisconsin Registry for Alzheimer’s Prevention (WRAP) were analyzed for cryptic HDGFL2 by MSD.

**Result:**

Sixty‐eight samples (25.6%) showed elevated levels of cryptic HDGFL2. These cryptic HDGFL2‐positive samples had a significantly higher proportion of phosphorylated‐tau/Aβ42‐positive cases (64.1%, p = 0.001). Twenty‐nine of the 107 individuals (27.1%) analyzed were positive for cryptic HDGFL2 at least once longitudinally. In 10 of the 29 (34.5%) individuals, cryptic HDGFL2 levels increased across each longitudinal sample collection, while they decreased longitudinally in 13 (44.8%). In six (20.7%), cryptic HDGFL2 levels displayed a peak during the longitudinal sampling. Twenty‐three of the 29 individuals had associated cognitive data. Eight individuals (34.8%) were from the group with increasing cryptic HDGFL2 levels, 11 (47.8%) from the group with decreasing levels, and four (17.4%) from the group displaying a peak. Each of these individuals was cognitively normal, besides one person in the decreasing group with Mild Cognitive Impairment (MCI). In this individual, cryptic HDGFL2 levels were already elevated prior to MCI diagnosis.

**Conclusion:**

The proportion of individuals with elevated cryptic HDGFL2 levels corresponds with the expected percentage of AD cases with co‐pathology of TDP‐43. Our findings of TDP‐43 dysfunction in many cognitively normal individuals suggest that TDP‐43 loss of function is an early event in disease that can precede symptom development.

